# Insecticidal activity, chemical characterization, and biochemical responses induced by selected essential oils against *Tuta absoluta* (Lepidoptera: Gelechiidae)

**DOI:** 10.1038/s41598-026-63750-2

**Published:** 2026-07-30

**Authors:** Hanaa E. Sadek, Enas Adel Abd-Elatef

**Affiliations:** 1 Department of Pests and Plant Protection, Agricultural and Biological Research Institute, National Research Centre, Cairo, 12622 Egypt; 2 Plant Protection Research Institute, Agricultural Research Center (ARC), Giza, Egypt

**Keywords:** *Tuta absoluta*, Essential oils, Citronella, Geranium, LC_50_, Enzymatic activity, Oxidative stress, Botanical insecticides, Biochemistry, Biological techniques, Biotechnology, Chemical biology, Plant sciences, Zoology

## Abstract

The tomato leaf miner, *Tuta absoluta* (Meyrick) (Lepidoptera: Gelechiidae), is a major pest causing severe damage to tomato crops worldwide. The present study evaluated the insecticidal efficacy of *Cymbopogon nardus* (citronella) and *Pelargonium graveolens* (geranium) essential oils against *T. absoluta* larvae and their impact on selected biochemical and oxidative stress parameters under laboratory conditions. GC-MS analysis identified citral and related monoterpenes as the major components of citronella oil, while geranium oil was rich in citronellol. Toxicity assays demonstrated dose-dependent larval mortality for both oils, with citronella oil exhibiting slightly higher potency (LC_50_ = 3.12%; LC_90_ = 6.37%) than geranium oil (LC_50_ = 3.88%; LC_90_ = 7.65%). Biochemical analyses revealed significant reductions in GABA-transaminase activity, total protein, and total lipid contents in treated larvae, indicating disruption of neurotransmission, metabolism, and energy reserves. Furthermore, oxidative stress markers showed decreased total antioxidant capacity and superoxide dismutase activity, together with increased lipid peroxidation, indicating the induction of oxidative stress in treated larvae. These findings demonstrate that both essential oils possess significant insecticidal activity and induce marked biochemical and oxidative stress responses in *T. absoluta* larvae. Therefore, citronella and geranium essential oils may represent promising eco-friendly botanical insecticides for incorporation into integrated pest management programs for tomato crops.

**Supplementary Information:**

The online version contains supplementary material available at 10.1038/s41598-026-63750-2.

## Introduction

Tomato (*Solanum lycopersicum* L.) is one of the world’s most economically important vegetable crops, cultivated extensively under both open-field and protected cultivation systems for domestic consumption and international markets. Owing to its high nutritional value, tomatoes are an excellent source of vitamins, minerals, carotenoids, flavonoids, phenolic compounds, and antioxidants, particularly lycopene, which has been associated with antioxidant and anticancer properties. Consequently, tomato production plays a vital role in global food security and agricultural economies^1–3^.

Among the major constraints affecting tomato production, the tomato leaf miner, *Tuta absoluta* (Meyrick) (Lepidoptera: Gelechiidae), is considered one of the most destructive invasive pests worldwide. Since its introduction into Africa and the Middle East, including Egypt, this pest has caused severe economic losses through extensive feeding damage on leaves, stems, flowers, buds, and fruits, with crop losses reaching up to 100% under severe infestations^4,5^. In addition to tomato, *T. absoluta* attacks several cultivated and wild Solanaceous hosts, including potato, eggplant, and black nightshade, thereby increasing its ecological adaptability and economic importance^6,7^.

Synthetic insecticides remain the primary approach for controlling *T. absoluta*. However, their intensive and repeated use has resulted in the rapid evolution of resistance to several insecticide groups, including avermectins, spinosyns, pyrethroids, organophosphates, and oxadiazines. Furthermore, extensive insecticide application negatively affects beneficial arthropods, natural enemies, pollinators, and the surrounding environment, thereby compromising the sustainability of pest management programs^8,9^. Recent reports continue to identify insecticide resistance as one of the major obstacles limiting effective management of *T. absoluta*, emphasizing the urgent need for environmentally sustainable alternatives within integrated pest management (IPM) programs^10–12^.

Integrated pest management strategies combining biological control agents, pheromone-based monitoring, cultural practices, host plant resistance, and botanical insecticides have therefore gained increasing attention as sustainable alternatives to conventional chemical control. Predatory mirid bugs such as *Macrolophus pygmaeus* and *Dicyphus spp*., entomopathogenic microorganisms, resistant tomato cultivars, and pheromone trapping systems have demonstrated promising results in suppressing *T. absoluta* populations while minimizing environmental impacts^10,13,14^.

Among eco-friendly alternatives, plant essential oils have emerged as promising botanical insecticides because of their biodegradability, relatively low mammalian toxicity, and complex mixtures of biologically active secondary metabolites. Essential oils exhibit multiple modes of action, including insecticidal, fumigant, repellent, antifeedant, ovicidal, and growth-disrupting activities, which reduce the likelihood of resistance development compared with conventional insecticides^15–18^. Their biological activities are mainly attributed to oxygenated monoterpenes and sesquiterpenes that interfere with insect nervous, metabolic, and physiological processes.

Among these botanical products, *Cymbopogon nardus* (citronella) essential oil and *Pelargonium graveolens* (geranium) essential oil have attracted considerable attention because of their high contents of oxygenated monoterpenes with proven insecticidal properties. Citronella oil is rich in citronellal, citral, and geraniol, whereas geranium oil contains high levels of citronellol and geraniol. These compounds have demonstrated toxic, repellent, and feeding-deterrent activities against numerous agricultural insect pests through interference with neurotransmission, membrane integrity, detoxification pathways, and oxidative balance^19–21^. Nevertheless, despite the growing body of evidence supporting their insecticidal efficacy, information regarding their biochemical effects on *T. absoluta*, particularly their influence on neurotransmission-related enzymes and oxidative stress biomarkers, remains limited.

Biochemical biomarkers provide valuable information for understanding the physiological responses of insects to botanical insecticides. Alterations in gamma-aminobutyric acid transaminase (GABA-T), antioxidant defense enzymes, lipid peroxidation, and other metabolic indicators may reflect disturbances in neuronal function, oxidative balance, and energy metabolism following exposure to bioactive phytochemicals^22^. However, studies integrating toxicity assessment with biochemical and oxidative stress analyses of essential oils against *T. absoluta* are still scarce.

Therefore, the present study aimed to evaluate the insecticidal efficacy of *Cymbopogon nardus* and Pelargonium graveolens essential oils against *Tuta absoluta* larvae, characterize their chemical composition using GC–MS analysis, and investigate their effects on selected biochemical and oxidative stress biomarkers. The findings provide further insight into the physiological responses of *T. absoluta* to these botanical insecticides and support their potential application as environmentally friendly components of integrated pest management programs.

## Materials and methods

### Insect rearing

A laboratory colony of *Tuta absoluta* was established from larvae collected from naturally infested tomato fields in [Nubaria region, Egypt]. Larvae were reared on healthy leaves of tomato (*Solanum lycopersicum* L., cv. Castle Rock) under controlled laboratory conditions at 25 ± 2 °C, 65 ± 5% relative humidity, and a 16:8 h (light: dark) photoperiod. The colony was maintained for [two/three] laboratory generations before initiating the bioassays to minimize field-related variability. Prior to each experiment, larvae were synchronized by selecting healthy individuals of the same developmental stage (second instar) and similar size to ensure uniformity among treatments.

The larvae were raised and preserved on tomato leaves and grown in plastic containers inside a glass cage (50 × 50x100 cm3), 10 larvae were placed in each replicates, ten replicates were made for each concentration, tomato leaves were sprayed at each concentration, and Mortality was initially assessed 24 h after treatment and subsequently recorded every 24 h for five days. However, LC_50_ and LC_90_ values were estimated by probit analysis using the mortality data obtained 72 h after treatment, as this exposure period provided the most reliable dose–response relationship. For biochemical and oxidative stress analyses, surviving larvae were exposed to the LC_50_ concentration of the tested formulation, as determined by the 72-h bioassay.

### Plant materials and chemicals

Citronella *Cymbopogon nardus* (L.) and Geranium *Pelargonium graveolens (Thunb)* were obtained from the oil extraction unit at the National Research Centre in Cairo, Egypt. A Clevenger-type device was used for four hours to extract the essential oils from 100 g of dried leaves. The water and oil-containing condensate was gathered and placed in a flask with sodium chloride. Diethyl ether was then used to remove the condensate. After adding anhydrous sodium sulfate to eliminate moisture, the solvent was allowed to evaporate at room temperature under a nitrogen flow. Lastly, the materials were kept at −20 °C in airtight glass vials covered with aluminum foil until analysis.

### Gas chromatography-mass spectrometry (GC-MS)

The sample was dissolved in dichloromethane and injected into GC**.** The GC-MS system (Agilent Technologies) was equipped with gas chromatograph (7890B) and mass spectrometer detector (5977A) at Central Laboratories Network, National Research Centre, Cairo, Egypt. The HP-5MS column (15 m × 0.25 mm internal diameter and 0.25 μm film thickness) was installed in the GC. Hydrogen was used as the carrier gas in the analyses, with a flow rate of 1.1 ml/min at a split (1:10), an injection volume of 1.0 µl, and the following temperature program: 40 °C for one minute; rising at 10 °C/min to 200 °C and held for one minute; rising at 20 °C/min to 220 °C and held for one minute; rising at 30 °C/min to 300 °C and held for three minutes. At 250 and 300 degrees Celsius, the injector and detector were maintained. Electron ionization (EI) at 70 eV was used to obtain mass spectra, with a solvent delay of 1.60 min and a spectral range of m/z 33–600. The quad temperature was 150 °C and the mass temperature was 230 °C. By comparing the spectrum fragmentation pattern with data from the Wiley and NIST Mass Spectral Library, many substances were identified^23^.

### Insecticidal activity of the essential oils

Two oils were used for examination: Citronella oil *Cymbopogon nardus* (L.) and Geranium oil, *Pelargonium graveolens (Thunb.)* were prepared by mixing each of the tested oils in five different concentrations (0.625, 1.25, 2.5, 5 and 7.5%) with a few drops of emulsifier 0.1% (Tween 80) and distilled water^24^. The experiment involved placing tomato plant leaves in Petri dishes with ten *Tuta* larvae and spraying them with the prepared concentrations of emulsified essential oils. Essential oil concentrations were prepared as v/v solutions using distilled water containing Tween 80 as an emulsifying agent. The same concentration of Tween 80 was used in the control treatment to exclude any solvent effect on larval mortality.

The concentration range was selected based on preliminary range-finding bioassays to ensure adequate mortality for probit analysis and LC estimation.

Mortality was assessed at 24-h intervals for five consecutive days. The lethal concentration (LC) values were estimated using mortality data recorded after 72 h of exposure by probit analysis. Since mortality in the control group was 0%, Abbott’s formula was not applied, whenever control mortality exceeded 0%. Larvae that failed to respond to gentle stimulation with a fine camel-hair brush were considered dead.

### Enzymatic activity

#### Preparation of tissue homogenates

Crude enzyme sources were prepared by grinding a specific weight of organisms with liquid nitrogen and suspending them in cold sodium phosphate buffer (50 mM, pH 7) to reach the desired enzyme concentration. Homogenization was done in an Eppendorf tube using a manual homogenizer at around 4 °C. The extracts were then centrifuged at 10,000 × g for 10 min at 4 °C to eliminate any precipitate. Enzyme activities were measured in the clear supernatants obtained.

#### Concentration of total protein

Following bovine serum albumin (BSA) as a protein standard, the total protein was calculated following the method recommended by Bradford^25^.

#### Concentration of total lipids

It was color metrically assayed at 520 nm using the method demonstrated by^26^ following instruction of the commercially available kit obtained from Spectrum Diagnostics Egyptian Company for Biotechnology (Cairo, Egypt).

#### Activity of gamma amino butyric acid transaminase (GABA-T) enzyme

The method described by De Boer and Bruinvels^27^ with changes suggested by Pandey and Singh^28^ was used to measure GABA-T activity. In addition to 100 μL of 2 mM α-ketoglutarate (pH 7), 100 μL of 20 mM 2-mercaptoethanol, and 20 μL of 1.1 mM β-NAD, the reaction buffer contained 50 mM Tris–HCl (pH 8.5). 200 µL of 3 mM gamma-aminobutyric acid (GABA) was added to initiate the reaction. The absorbance was measured at 340 nm after 30 min of incubation at 25 °C.

### Markers of the oxidative stress

#### Total antioxidant capacity

It was assessed using the green phosphate/Mo5 + complex test in accordance with the procedure outlined by Prieto et al.^29^µmol Trolox Equivalents (TE)/g tissue was used to express the outcome.

#### Superoxide dismutase (SOD) activity

It was measured in the tissue homogenates using the technique recommended by Nishikimi^30^. Units per milligram of protein (U/mg protein/min) were used to define the results.

#### Lipid peroxidation product (LPO)

Malondialdehyde (MDA), the last byproduct of the lipid peroxidation cycle, was used to express the LPO. Thiobarbituric acid reactive substances (TBARs) were identified using the approach of^31^. The findings were reported as nmol/g tissue.

#### Total protein carbonyl content (TPC)

.The method outlined by Levine et al.^32^ was used to evaluate the Total protein carbonyl content TPC content. The findings were reported as nmol of reactive carbonyl compounds per mg of tissue protein.

### Statistical analysis

All biochemical data were expressed as mean ± standard error (SE) of ten independent replicates. Data were analyzed using one-way analysis of variance (ANOVA), followed by Tukey’s honestly significant difference (HSD) test for multiple comparisons. Differences among treatments were considered statistically significant at *P* < 0.05. Statistical analyses were performed using SPSS software (Version 18,). Different lowercase letters indicate significant differences among treatment.

## Results & discussion

**Fig. 1:**
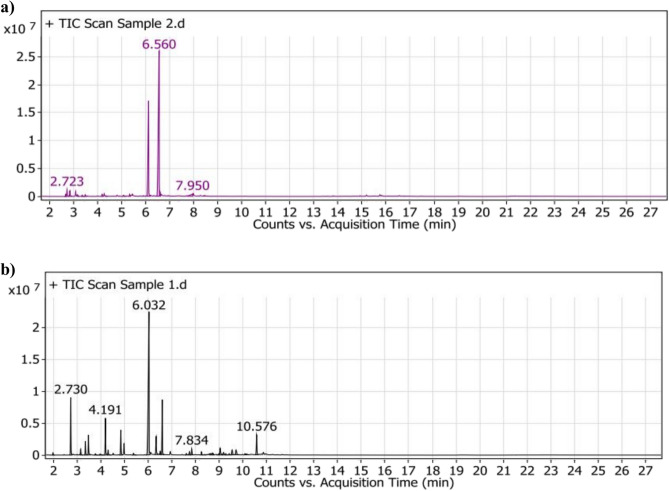
GC-MS chromatogram of (**a**) *Citronella*, and (**b**) *Geranium* essential oils.

The chemical composition of citronella essential oil was analyzed using GC-MS, and the identified compounds along with their retention times (RT) and relative percentages are presented in (Table 1, Fig. 1a) a total of 15 compounds were detected, representing the major constituents of the oil.

**Table 1:** GC-MS analysis of *Citronella* essential oil.

Peak	RT	Compound	Area Sum %
1	2.665	2,3-Dehydro-1,8-cineole	0.41
2	2.723	.beta.-Myrcene	2.06
3	2.846	2,6-Dimethyl-1,3,5,7-octatetraene, E,E-	1.29
4	3.161	Bicyclo[3.1.0]hexan-2-ol,2-methyl-5-(1-methylethyl)-,(1.alpha.,2.beta.,5.alpha.)-	0.56
5	3.354	.beta.-Ocimene	0.37
6	3.483	trans-.beta.-Ocimene	0.49
7	4.184	Linalool	0.6
8	4.339	1-Cyclohexene-1-carboxaldehyde, 4-(1-methylethenyl)-, (S)-	0.23
9	5.079	cis-Verbenol	0.5
10	5.33	Isoneral	0.55
11	6.115	Neral	29.97
12	6.56	2,6-Octadienal, 3,7-dimethyl-, (E)-	59.23
13	6.592	2-Isopropenyl-5-methylhex-4-enal	1.05
14	7.95	Cyclopropanecarboxaldehyde, 2-methyl-2-(4-methyl-3-pentenyl)-, trans-(. + -.)-	2.4
15	8.452	Cyclopentaneacetaldehyde, 2-formyl-3-methyl-.alpha.-methylene-	0.3

The results revealed that citronella oil is predominantly composed of oxygenated monoterpenes. The major compound identified was 2,6-Octadienal, 3,7-dimethyl-, (E)- (citral), which accounted for 59.23% of the total composition, followed by neral at 29.97%. Together, these two compounds constituted the vast majority of the oil composition.Other minor constituents were detected in relatively low percentages, including ß-myrcene (2.06%), cyclopropane carboxaldehyde derivatives (2.40%), and 2 isopropenyl-5-methylhex-4-enal (1.05%). Several additional compounds such as linalool (0.60%), cis-verbenol (0.50%), and ß-ocimene isomers were also present in trace amounts.

Overall, the results indicate that citronella oil is characterized by a high concentration of aldehydic monoterpenes, particularly citral and its isomers, which are known for their biological activity.

The GC-MS analysis demonstrated that citronella essential oil is rich in oxygenated monoterpenes, with citral (a mixture of geranial and neral) being the dominant component. The high proportion of citral (˜59%) along with neral (˜30%) is consistent with previous reports on the chemical composition of citronella oil, confirming its typical chemo type.

These compounds are well known for their potent insecticidal and repellent properties. Citral, in particular, has been reported to exhibit strong fumigant toxicity and neurotoxic effects against various insect pests, which may contribute significantly to the observed bioactivity of citronella oil in the present study. The presence of minor constituents such as ß-myrcene, linalool, and Ocimene may also play a synergistic role, enhancing the overall biological effectiveness of the oil.

Variations in the composition of essential oils are commonly influenced by several factors, including plant origin, environmental conditions, extraction methods, and plant developmental stage. Nevertheless, the dominance of citral and related compounds in the current analysis supports the potential use of citronella oil as an effective botanical insecticide.

From a biochemical perspective, the high content of bioactive monoterpenes may interfere with key physiological processes in insects, including enzyme activity. These compounds are known to affect detoxification enzymes and gamma amino butyric acid transaminase (GABA-T) enzyme activity, which could explain the enzymatic changes observed in treated insects in this study.

The chemical composition of geranium essential oil was analyzed using GC–MS, and the identified constituents along with their retention times and relative percentages are presented in (Table 2, Fig. 1b) a total of 33 compounds were identified, reflecting the complex nature of the oil.

**Table 2:** GC-MS analysis of *Geranium* essential oil.

Peak	RT	Compound	Area Sum %
1	1.977	.alpha.-Pinene	0.39
2	2.73	.beta.-Myrcene	8.75
3	3.148	Cyclohexene, 1-methyl-5-(1-methylethenyl)-, (R)-	1.19
4	3.348	.beta.-Ocimene	2.25
5	3.476	trans-.beta.-Ocimene	3.22
6	3.766	2-Furanmethanol, 5-ethenyltetrahydro-.alpha.,.alpha.,5-trimethyl-, cis-	0.28
7	3.985	3-Carene	0.41
8	4.191	Linalool	6.68
9	4.3	2H-Pyran, tetrahydro-4-methyl-2-(2-methyl-1-propenyl)-	0.98
10	4.841	L-Menthone	4.27
11	5.369	.alpha.-Terpineol	0.48
12	6.032	Citronellol	41.62
13	6.334	2,6-Octadien-1-ol, 3,7-dimethyl-, (Z)-	3.72
14	6.502	Neral	0.73
15	6.592	6-Octen-1-ol, 3,7-dimethyl-, formate	9.24
16	7.602	Bicyclo[4.1.0]heptane, 3,7,7-trimethyl-, (1.alpha.,3.alpha.,6.alpha.)-	0.39
17	7.738	Copaene	0.7
18	7.834	(-)-.beta.-Bourbonene	1.24
19	8.246	Caryophyllene	0.67
20	8.381	(1R,2S,6S,7S,8S)-8-Isopropyl-1-methyl-3-methylenetricyclo[4.4.0.02,7]decane-rel-	0.14
21	8.632	Alloaromadendrene	0.52
22	8.748	Aromandendrene	0.31
23	8.986	.beta.-Longipinene	0.39
24	9.038	Azulene, 1,2,3,3a,4,5,6,7-octahydro-1,4-dimethyl-7-(1-methylethenyl)-, [1R-(1.alpha.,3a.beta.,4.alpha.,7.beta.)]-	1.95
25	9.186	(3R,3aR,3bR,4S,7R,7aR)-4-Isopropyl-3,7-dimethyloctahydro-1H-cyclopenta[1,3]cyclopropa[1,2]benzen-3-ol	0.89
26	9.276	Naphthalene, 1,2,4a,5,6,8a-hexahydro-4,7-dimethyl-1-(1-methylethyl)-, (1.alpha.,4a.alpha.,8a.alpha.)-	0.3
27	9.418	.gamma.-Muurolene	0.34
28	9.54	cis-Calamenene	1.4
29	9.707	.beta.-Guaiene	1.7
30	10.087	Butanoic acid, 3,7-dimethyl-2,6-octadienyl ester, (E)-	0.19
31	10.119	1H-Cycloprop[e]azulen-7-ol, decahydro-1,1,7-trimethyl-4-methylene-, [1ar-(1a.alpha.,4a.alpha.,7.beta.,7a.beta.,7b.alpha.)]-	0.16
32	10.164	Ledene oxide-(II)	0.18
33	10.576	2-Naphthalenemethanol, 1,2,3,4,4a,5,6,7-octahydro-.alpha.,.alpha.,4a,8-tetramethyl-, (2R-cis)-	4.28

The results indicated that geranium oil is mainly composed of oxygenated monoterpenes and sesquiterpenes. The predominant compound was citronellol, representing 41.62% of the total composition. This was followed by butanoic acid, 3,7-dimethyl-2,6-octadienyl ester (E-) (9.24%), ß-myrcene (8.75%), and linalool (6.68%).

Other notable constituents included L-menthone (4.27%), 2-naphthalenemethanol derivatives (4.28%), and (Z)-2,6-octadien-1-ol (3.72%), while several minor components such as ß-ocimene isomers, caryophyllene, copaene, and muurolene were detected in lower concentrations.

Overall, the results demonstrate that geranium oil is rich in monoterpenoid alcohols, with citronellol being the dominant component, along with a diverse array of minor constituents contributing to its chemical profile. The GC-MS analysis revealed that geranium essential oil is characterized by a high proportion of oxygenated monoterpenes, particularly citronellol, which is widely recognized as one of the major bioactive constituents of geranium oil. The dominance of citronellol (41.62%) observed in the present study is consistent with previous reports describing geranium oil chemotypes rich in monoterpenoid alcohols.

GC-MS analysis revealed that *Pelargonium graveolens* essential oil was dominated by citronellol together with other oxygenated monoterpenes and minor terpenoid constituents. Previous studies have demonstrated that essential oils rich in citronellol and related monoterpenes exhibit significant insecticidal and behavioral effects against several insect pests through multiple physiological targets rather than a single specific mode of action. Recent investigations have highlighted the importance of oxygenated monoterpenes in disrupting normal insect physiology, contributing to mortality, growth inhibition, and alterations in biochemical parameters^33^.

The insecticidal activity observed in the present study is therefore likely associated with the combined action of citronellol and other constituents, including linalool, menthone, ß-myrcene, ocimene isomers, and sesquiterpenes. Increasing evidence suggests that the biological efficacy of essential oils results from complex interactions among their components, where minor constituents may enhance penetration, stability, or target-site interactions of major compounds, leading to synergistic effects. Such synergism has been reported as a key factor underlying the effectiveness of botanical insecticides against lepidopteran pests^33–35^.

The biochemical responses recorded in Tuta absoluta larvae following treatment with geranium oil further support the occurrence of physiological disturbances induced by the oil constituents. Significant reductions in GABA-T activity, total protein, and total lipid contents indicate disruption of normal metabolic processes. However, these biochemical changes should be interpreted cautiously, as alterations in a single enzyme or metabolite cannot conclusively identify a specific mode of action. Essential oils are known to interact with multiple neurophysiological and metabolic targets, including neurotransmitter systems, ion channels, and detoxification pathways. Therefore, the observed responses are more appropriately considered indicators of metabolic and physiological stress rather than definitive evidence of a particular toxicological mechanism^36^.

Similarly, the reductions observed in antioxidant-related parameters suggest a disturbance in the oxidative balance of treated larvae. Such responses may reflect impairment of antioxidant defenses and increased physiological stress following exposure to essential oil constituents. Comparable biochemical alterations have been reported in insects exposed to plant-derived essential oils, supporting the view that these natural products can affect several interconnected metabolic pathways simultaneously^34–36^.

Overall, the present findings indicate that the toxicity of *P. graveolens* essential oil against *T. absoluta* is likely attributable to the collective action of multiple constituents acting on different physiological targets. This multifaceted mode of action may reduce the likelihood of rapid resistance development and supports the potential use of geranium oil as a component of environmentally compatible pest management programs.

## Means followed by the same letter per column do not differ by Tukey test (*P* < 0.05). Sig. : (Significant)

The mean mortality percentages (± SE) of *Tuta absoluta* larvae exposed to different concentrations of citronella (*Cymbopogon nardus*) and geranium (*Pelargonium graveolens*) oils are presented in (Table 3). In general, mortality increased significantly with increasing concentrations of both oils, demonstrating a clear dose-dependent insecticidal effect (*p* < 0.001).

**Table 3:** *Tuta absoluta* exposed to varying quantities of the studied oils, geranium and citronella, had a mean death percentage (%) ± SE.

Conc	0.625%	1.25%	2.5%	5%	7.5%
(%) Treatments					
*Mortality% ± SE*					
*Cymbopogon nardus*	23.33 ± 3.33 a	33.33 ± 3.33 a	36.66 ± 3.33 a	73.33 ± 6.66 a	96.66 ± 3.33 a
*Pelargonium graveolens*	20.00 ± 0.00 a	26.66 ± 3.33 a	33.33 ± 3.33a	53.33 ± 6.66 b	93.33 ± 3.33a
Control	0.00 ± 0.00 b	0.00 ± 0.00 b	0.00 ± 0.00 b	0.0 ± 0.0 c	0.0 ± 0.0 b
F	12.5	9.00	20.25	6.25	182.2
Sig	0.00	0.00	0.00	0.00	0.00

At the lowest concentration (0.625%), mortality rates were 23.33 ± 3.33% for citronella and 20.00 ± 0.00% for geranium oil. Moderate increases were observed at 1.25% and 2.5%, with citronella consistently showing slightly higher toxicity. At 5%, citronella oil induced 73.33 ± 6.66% mortality, whereas geranium oil caused 53.33 ± 6.66% mortality. At the highest concentration (7.5%), nearly complete mortality was recorded for both oils (96.66 ± 3.33% for citronella and 93.33 ± 3.33% for geranium). The control treatment exhibited negligible mortality (0%), indicating that the observed effects were attributable to the essential oils. These differences were statistically significant at all concentrations (ANOVA, *p* < 0.001).The results indicate that both citronella and geranium essential oils possess significant insecticidal activity against *Tuta absoluta* larvae, aligning with previous research on botanical insecticides^17,37^. The dose-dependent mortality trend observed here is consistent with findings on other stored-product and field pests treated with plant essential oils^38,39^.

The comparatively higher mortality induced by citronella oil at intermediate concentrations may be related to its high content of citral and related aldehydes, which have been reported to exhibit strong fumigant and neurotoxic effects on insects^40,41^. Citral has been shown to disrupt insect neurotransmission and metabolic pathways, contributing to increased lethality^42,43^.

Geranium oil, dominated by citronellol and other oxygenated monoterpenes, also demonstrated potent bioactivity at higher concentrations. Citronellol has been documented for its contact toxicity and repellent effects against various insect pests^44,45^. The presence of linalool, Menthone, and other minor constituents may contribute synergistically, enhancing overall efficacy^46,47^.

The significant insecticidal activity of both oils supports their potential utility as eco-friendly alternatives to synthetic insecticides for *T. absoluta* management, particularly in the context of increasing resistance to conventional chemicals^9,48^. The nearly complete mortality observed at the highest concentration (7.5%) suggests that these oils can provide effective control when applied at adequate doses, but further studies on field efficacy and formulation optimization are warranted^37,38^.

Similarly, LC_95_ and LC_99_ values followed the same trend, with *C. nardus* exhibiting lower lethal concentrations compared to *P. graveolens*. The slope values (± SE) were 0.394 ± 0.051 and 0.340 ± 0.046, respectively, indicating relatively steep dose–response relationships. Chi-square values were non-significant (*p* ≤ 0.05) for both oils, suggesting a good fit of the probit model to the observed mortality data. The probit analysis indicates that both citronella (*C. nardus*) and geranium (*P. graveolens*) essential oils exhibit potent insecticidal activity against *Tuta absoluta* larvae under laboratory conditions. The lower LC_50_ and LC_90_ values recorded for *C. nardus* compared to *P. graveolens* suggest that citronella oil is more toxic to *T. absoluta* at equivalent concentrations, which is in agreement with the higher mortality percentages observed in the bioassay results (Table 4).

**Table 4:** Toxicity of *C. nardus* and *P .graveolens* on *T. absoluta* after 72 h of treatment.

	*Cymbopogon nardus*	*Pelargonium graveolens*
LC _50_	3.129(2.57–3.77)	3.88 (3.24–4.66)
LC _90_	6.37 (5.44–7.85)	7.656 (6.512–9.512)
LC _95_	7.29 (6.2–9.06)	8.726 (7.38–10.94)
LC _99_	9.026 (7.606–11.36)	10.73(9.002–13.635)
Slope ± SE	0.394 ± 0.051	0.34 ± 0.046
Chi-Square	7.07	7.834
Degree of freedom (df)	4	4
*P*-value	0.132b	0.09b

The results of probit analysis for the toxicity of *C. nardus* and *P. graveolens* essential oils against *T. absoluta* after 72 h of exposure are presented in (Table 4). Both oils demonstrated significant toxic effects, with LC_**50**_ values of 3.129% (95% CI 2.57–3.77) for *C. nardus* and 3.88% (95% CI 3.24–4.66) for *P. graveolens*. The LC_90_ values were 6.37% (95% CI 5.44–7.85) and 7.656% (95% CI 6.512–9.512) for *C. nardus* and *P. graveolens*, respectively.

The LC_50_ values obtained in this study are comparable to those reported for other botanical insecticides against lepidopteran pests, where essential oils and their constituents have demonstrated effective control at similar concentration ranges^17,37^. Lower LC_50_ values indicate higher potency, and the results here align with previous findings that oils rich in monoterpenoid aldehydes and alcohols often exhibit stronger toxicity^38,43^.

The presence of chemical constituents such as citral (major component in *C. nardus*) and citronellol (dominant in *P. graveolens*) may explain differences in toxicity. Citral has been documented to affect insect nervous systems and respiratory metabolism, leading to enhanced lethality in several insect species^40^. Similarly, citronellol has shown insecticidal and repellent properties, though often requiring slightly higher concentrations to achieve comparable mortality levels^44,45^.

The non-significant chi-square values indicate that the probit model adequately describes the dose–response relationships, confirming the reliability of the LC estimates. Comparable studies on essential oils against *T. absoluta* and related pests have also reported well-fitting probit models with non-significant chi-square values^39,47^.

From an applied perspective, the lower LC values for citronella oil suggest its greater potential as an eco-friendly alternative for managing *T. absoluta* in tomato production systems. Botanical insecticides with effective toxicity at low concentrations are particularly valuable due to their reduced environmental impact and lower risk of resistance development^9,17^.

Values are presented as mean ± SE. Means followed by different letters within the same column are significantly different according to Tukey’s Multiple Range Test at P ≤ 0.05. (F values) are presented for each enzyme.

The effects of *Cymbopogon nardus* and *Pelargonium graveolens* essential oils on *Tuta absoluta* enzymatic activity and biochemical constituents are presented in (Table 5).

**Table 5:** Enzymatic activity.

	Gamma amino butyric acid transaminase (GABA-T) (mg/L)	Total protein	Total lipids
		(mg/gm tissue)	
*Cymbopogon nardus*	3.19 ± 0.02 b	2.28 ± 0.02b	0.97 ± 0.03b
*Pelargonium graveolens*	2.98 ± 0.02c	2.13 ± 0.02c	0.90 ± 0.03c
Control	4.08 ± 0.02 a	2.92 ± 0.02a	1.24 ± 0.04a
F	908.17	362.9	396.2

	TAC (µmol trolox equivalents (TE)/g tissue)	SOD (units/g tissue)	LPO (nmol/g tissue)	TPC (nmol/ mg protein)
*Cymbopogon nardus*	12.46 ± 0.11 b	1.06 ± 0.05 b	6.71 ± 0.05 b	4.59 ± 0.11b
*Pelargonium graveolens*	11.64 ± 0.10 c	0.99 ± 0.04 c	7.19 ± 0.06 a	4.92 ± 0.11a
Control	15.95 ± 0.14 a	1.36 ± 0.06 a	5.24 ± 0.04 c	3.59 ± 0.08 c
F	51,891	1904	3785	3187

GABA-T activity was significantly reduced in treated larvae compared to the control. *C. nardus* treatment led to 3.19 ± 0.02 mg/L, while *P. graveolens* caused 2.98 ± 0.02 mg/L, compared to 4.08 ± 0.02 mg/L in the untreated control.

Total protein content decreased in response to essential oil treatments, with values of 2.28 ± 0.02 mg/g tissue for *C. nardus* and 2.13 ± 0.02 mg/g tissue for *P. graveolens*, relative to 2.92 ± 0.02 mg/g tissue in the control.

Total lipid content also declined in treated larvae, reaching 0.97 ± 0.03 mg/g for *C. nardus* and 0.90 ± 0.03 mg/g for *P. graveolens*, compared to 1.24 ± 0.04 mg/g in the control group.

These results indicate that both essential oils induce significant biochemical alterations in *T. absoluta* larvae, reflecting potential disruptions in neurotransmission, protein metabolism, and lipid homeostasis.

The observed reduction in GABA-T activity may indicate a disturbance in GABA metabolism following exposure to the essential oils. However, GABA-T activity alone is insufficient to establish inhibition of the GABAergic system. Essential oils are known to affect multiple neurophysiological targets, including acetylcholinesterase, GABA receptors, octopamine receptors, and ion channels. Therefore, the present findings should be interpreted as evidence of a possible involvement of GABA metabolism rather than definitive proof of the underlying neurotoxic mechanism. Further biochemical and molecular studies are required to confirm the precise mode of action. suggest that both citronella and geranium oils interfere with the GABAergic system of *Tuta absoluta* larvae. GABA-T plays a critical role in the catabolism of aminobutyric acid (GABA), a major inhibitory neurotransmitter in insects, and its inhibition may result in neuronal hyper excitation, impaired locomotion, and mortality^49,50^. The slightly higher GABA-T activity in citronella-treated larvae compared to geranium may reflect differences in the neuroactive constituents of the oils, such as citral versus citronellol^17,37^^**.**^

The significant decrease in total protein content indicates that both oils negatively impact protein synthesis and metabolism, which could contribute to impaired growth and development of the larvae^34,47^. Similarly, the reduction in total lipids suggests interference with lipid metabolism, possibly affecting energy reserves and membrane integrity, which are essential for larval survival^38^.

These biochemical disruptions are likely linked to the major components identified in the oils. Citral, a predominant compound in *C. nardus*, has been reported to disrupt enzyme activities in insects, including detoxifying and neurotransmitter-related enzymes^40^. Citronellol, dominant in *P. graveolens*, has also shown effects on enzymatic and metabolic pathways, although slightly less pronounced than citral, which may explain the minor differences observed between the two oils.

Overall, these findings complement the mortality data and LC_50_ results (Tables 3 and 4), confirming that the essential oils act both as direct toxicants and as modulators of critical biochemical pathways in *T. absoluta* larvae. This dual mode of action reinforces their potential as eco-friendly botanical insecticides.

The effects of *C. nardus and P. graveolens* essential oils on oxidative stress markers in *T.absoluta* larvae are summarized in the table. Compared to the control group, larvae treated with essential oils exhibited significant alterations in antioxidant and oxidative stress parameters:

Total Antioxidant Capacity (TAC) decreased to 12.46 ± 0.11 µmol TE/g tissue (*C. nardus*) and 11.64 ± 0.10 µmol TE/g tissue (*P. graveolens*) relative to 15.95 ± 0.14 in the control.

Superoxide Dismutase (SOD) activity was reduced to 1.06 ± 0.05 units/g (*C. nardus*) and 0.99 ± 0.04 units/g *(P. graveolens*) versus 1.36 ± 0.06 in controls.

Lipid peroxidation (LPO) levels increased in treated larvae (6.71 ± 0.05 nmol/g for *C. nardus*; 7.19 ± 0.06 nmol/g for P. graveolens) compared with 5.24 ± 0.04 nmol/g in the control.

Total protein content (TPC) showed an increase to 4.59 ± 0.11 nmol/mg protein (*C. nardus*) and 4.92 ± 0.11 nmol/mg protein (*P. graveolens*), relative to 3.59 ± 0.08 in the control group.

These results indicate that essential oil treatments disrupt the antioxidant defense system of *T. absoluta,* leading to elevated oxidative stress.

The observed reductions in TAC and SOD activities suggest an oxidative imbalance and a possible impairment of the antioxidant defense system following exposure to the essential oils. However, these parameters alone are insufficient to confirm oxidative stress, and additional oxidative stress biomarkers would be required to establish this mechanism conclusively. However, these parameters alone are insufficient to confirm oxidative stress, and additional oxidative stress biomarkers would be required to establish this mechanism conclusively. SOD is a key enzyme in detoxifying superoxide radicals, and its suppression can result in accumulation of reactive oxygen species (ROS), leading to cellular damage^49,50^.

The significant increase in LPO levels confirms enhanced lipid peroxidation, indicating oxidative damage to cell membranes, which can impair larval metabolism and contribute to mortality^45,47^. The observed rise in TPC may reflect a stress-induced phenolic response, where larvae attempt to counteract oxidative stress by mobilizing phenolic compounds, though insufficient to prevent ROS-mediated damage.

These biochemical changes align with the reductions in GABA-T, total protein, and total lipid contents observed in treated larvae (Table 5), indicating that essential oils not only act as direct toxicants but also disrupt metabolic and oxidative homeostasis. Citral in *C. nardus* and citronellol in *P. graveolens* are likely responsible for these effects, consistent with their known bioactivity against insect enzymatic and antioxidant systems^40,44^.

Overall, the disruption of oxidative balance, coupled with neurotoxic and metabolic interference, suggests a multi-target mode of action for these essential oils, reinforcing their potential as eco-friendly insecticides against *T. absoluta*.

## Conclusion

The present study demonstrates that the essential oils of citronella and geranium exhibit significant insecticidal activity against *Tuta absoluta* larvae under laboratory conditions. GC-MS analysis revealed that citronella oil is rich in citral and related monoterpenes, while geranium oil contains high levels of citronellol, both of which are likely responsible for the observed toxic effects. Bioassays showed that both oils caused dose-dependent mortality, with citronella oil being slightly more potent, as evidenced by lower LC_50_, LC_90_, and LC_95_ values. In addition to direct toxicity, both essential oils significantly affected larval biochemical parameters, including reductions in GABA-T activity, total protein, and total lipid content, indicating interference with neurotransmission, metabolic processes, and energy reserves. Moreover, the oils disrupted the oxidative balance of *T. absoluta* larvae, as reflected by decreased total antioxidant capacity and SOD activity, coupled with increased lipid peroxidation and altered total protein content. These changes suggest that the oils exert their effects through multiple mechanisms, combining neurotoxicity, metabolic disruption, and oxidative stress. Overall, *C. nardus and P. graveolens* essential oils have demonstrated promising potential as eco-friendly botanical insecticides for the management of *T. absoluta*. Their multi-target mode of action, low required concentrations, and natural origin make them suitable candidates for integrated pest management strategies in tomato production systems, reducing reliance on synthetic chemicals and minimizing environmental impact.

## Supplementary Information

Supplementary Information 1.

## Data Availability

All data used in this study are available by email request to the corresponding author (hanyean2005@yahoo.com).
