# Supplementary material for: Insecticidal activity, chemical characterization, and biochemical responses induced by selected essential oils against Tuta absoluta (Lepidoptera: Gelechiidae)
Source: Sci Rep. 2026 Jul 30;16:23652. doi: 10.1038/s41598-026-63750-2 (PMC13424309; doi:10.1038/s41598-026-63750-2)
Supplement: Supplementary file 1 — Supplementary Information 1. [file 41598_2026_63750_MOESM1_ESM.doc]

|  |  | **Gamma amino butyric acid transaminase (GABA-T) (**mg/L**)** | **Total Protein** | **Total Lipids** |
| --- | --- | --- | --- | --- |
|  |  |  | **(**mg/gm tissue**)** | |
| **Control** | **1** | 4.05 | 2.88 | 1.18 |
|  | **2** | 4.12 | 2.93 | 1.23 |
|  | **3** | 4.07 | 2.95 | 1.31 |
|  | **Mean** | **4.08** | **2.92** | **1.24** |
|  | **SD** | **0.04** | **0.04** | **0.07** |
|  | **SE** | **0.02** | **0.02** | **0.04** |
| **Geranium** | **1** | 2.96 | 2.10 | 0.86 |
|  | **2** | 3.01 | 2.14 | 0.90 |
|  | **3** | 2.97 | 2.15 | 0.96 |
|  | **Mean** | **2.98** | **2.13** | **0.90** |
|  | **SD** | **0.03** | **0.03** | **0.05** |
|  | **SE** | **0.02** | **0.02** | **0.03** |
| **Citronella** | **1** | 3.17 | 2.25 | 0.92 |
|  | **2** | 3.22 | 2.29 | 0.96 |
|  | **3** | 3.18 | 2.30 | 1.02 |
|  | **Mean** | **3.19** | **2.28** | **0.97** |
|  | **SD** | **0.03** | **0.03** | **0.05** |
|  | **SE** | **0.02** | **0.02** | **0.03** |

|  |  | **TAC**  **(**TE/g tissue**)** | **SOD**  **(**units/g tissue**)** | **LPO**  **(**nmol/g tissue**)** | **TPC**  **(**nmol/ mg protein**)** |
| --- | --- | --- | --- | --- | --- |
| **Control** | **1** | 15.75 | 1.48 | 5.17 | 3.75 |
|  | **2** | 16.21 | 1.29 | 5.26 | 3.48 |
|  | **3** | 15.89 | 1.31 | 5.31 | 3.53 |
|  | **Mean** | **15.95** | **1.36** | **5.24** | **3.59** |
|  | **SD** | **0.24** | **0.10** | **0.07** | **0.14** |
|  | **SE** | **0.14** | **0.06** | **0.04** | **0.08** |
| **Geranium** | **1** | 11.50 | 1.08 | 7.08 | 5.14 |
|  | **2** | 11.83 | 0.95 | 7.20 | 4.77 |
|  | **3** | 11.60 | 0.96 | 7.28 | 4.84 |
|  | **Mean** | **11.64** | **0.99** | **7.19** | **4.92** |
|  | **SD** | **0.17** | **0.07** | **0.10** | **0.20** |
|  | **SE** | **0.10** | **0.04** | **0.06** | **0.11** |
| **Citronella** | **1** | 12.30 | 1.15 | 6.61 | 4.80 |
|  | **2** | 12.66 | 1.01 | 6.73 | 4.46 |
|  | **3** | 12.41 | 1.02 | 6.80 | 4.52 |
|  | **Mean** | **12.46** | **1.06** | **6.71** | **4.59** |
|  | **SD** | **0.18** | **0.08** | **0.09** | **0.18** |
|  | **SE** | **0.11** | **0.05** | **0.05** | **0.11** |
